# Oppression, liberation, wellbeing, and ecology: organizing metaphors for understanding health workforce migration and other social determinants of health

**DOI:** 10.1186/s12992-018-0397-y

**Published:** 2018-08-09

**Authors:** Akhenaten Benjamin Siankam Tankwanchi

**Affiliations:** DST/NRF SARChi Programme on the Health Workforce, School of Public Health, Faculty of Health Sciences, University of the Witswatersrand, Johannesburg, South Africa

**Keywords:** Oppression, Liberation, Empowerment, Wellbeing, Interdisciplinary, Transdisciplinary, Ecological model, Psychopolitical validity, Health inequities, Social determinants of health, Health workforce migration

## Abstract

**Background:**

The Commission on Social Determinants of Health (CSDH) identifies the maldistribution of power, money, and resources as main drivers of health inequities. The CSDH further observes that tackling these drivers effectively requires interventions to focus at local, national, and global levels. Consistent with the CSDH’s observation, this paper describes the eco-psychopolitical validity (EPV) paradigm, a multilevel and transdisciplinary model for research and action, thus far insufficiently tapped, but with the potential to systematize the exploration of the social determinants of health.

**Results:**

Using the physician migration from Sub-Saharan Africa (SSA) to the United States as illustration, this paper articulates how the EPV model can be applied to the systematic analysis of a complex social problem with health inequity implications. To help explore potential determinants of physician migration, a comprehensive coding matrix is developed; with the organizing metaphors of the EPV model–namely oppression, liberation, and wellbeing–serving as analytical categories. Through the lens of the EPV model, migrating physicians are revealed as both ecological subjects enmeshed in a vast web of transnational processes linking source and destination countries, and potential change agents pursuing liberation and wellbeing. While migration may expand the opportunities of émigré physicians, it is argued that, the pursuit of wellbeing by way of migration cannot fully materialize abroad without some efforts to return home, physically or socially.

**Conclusion:**

Clarifying the relationship between various social determinants of health and health inequities at different levels of analysis is a more complex but essential endeavor to knowledge generation than using a one-dimensional frame. With its roots in interdisciplinary thinking and its emphasis on both individual and contextual factors, the EPV paradigm holds promise as a model for examining the social determinants of health.

## Background

It is a world in which a Ghanaian-born student at Harvard Medical School might return to Ghana to do a summer of Harvard-sponsored research on the causes of the brain drain affecting the country of her birth, and no cognitive dissonance ensues; a world in which recent events and processes are speeding (or slowing) the emergence and retrenchment of social inequalities almost invisible to some who easily traverse national boundaries, while the same social pathologies are glaringly obvious to others who share, however fleetingly, the same social field. It is a world in which … [highly skilled health workers and epidemics like HIV/AIDS, Ebola, and Zika transcend borders] while the fruits of science, including treatment, are blocked at customs [1].

The 2014 and deadliest Ebola epidemic to date, visited upon the West African countries of Guinea, Liberia, and Sierra Leone exposed the calamitous state of health systems in the affected countries. Over 11,300 Ebola-infected persons perished, including many healthcare providers [2]. Gostin has argued that “the real reasons the outbreak turned into a tragedy of such proportions are human resource shortages and fragile health systems” [3]. Fixing these “inherent structural deficiencies” will necessarily involve addressing the intractable issue of health workforce migration from the region. Although the true magnitude of this ‘brain drain’ is difficult to quantify due to limitations from data collection, data obsolescence, and data restrictions [4–6], theoretical attempts to explain why and how it occurs are just as daunting [7]. Merely juxtaposing a list of push factors in source countries and pull factors from receiving countries, as commonly found in the health policy literature, provides little insight as to how medical migration occurs and why it persists despite widespread knowledge of the inequalities of medical personnel between doctor-outsourcing but need-plagued countries and doctor-receiving nations [8]. Moreover, such binary analysis does not inform as to why some health professionals with ample opportunities to emigrate choose instead to stay in their resource-limited homelands.

Consider, as a case in point, 52 years old Ghanaian identical twin brothers and physicians Dr. Atsu and Dr. Etse.^1^ They graduated from high school with honors in the early 1980’s and were among a select few from a highly competitive national pool of Ghanaian high school graduates admitted to pursue an engineering degree in Europe. They both declined the offer and chose instead to attend a local medical school in Ghana, from which they both graduated in 1990. Both are married and fathers of three [9].

In 1997, Atsu left Ghana to reunite with his wife in Canada before relocating to the United States (USA) to complete Graduate Medical Education (GME) residency training. He currently holds a faculty position in a medical school in the greater Washington, DC metropolitan area. Conversely, Etse opted to stay and practice in a public hospital in Ghana despite the encouragements of his twin brother to follow him to the USA. He cited his concerns of becoming a “second-class citizen” in a foreign country and his unwillingness to go through the hassles of residency admission in the USA as significant factors in his decision to stay in Ghana. “There is no place like home,” he said repeatedly during our interview [9]. What sets these men apart today is not merely time-space distanciation, nor the individuation which may have occurred as a result of their geographic separation [10]. What mainly differentiates them is their everyday practice in two greatly unequal health systems. Etse practices in a heavily indebted poor country (HIPC) with critical shortages of resources [11]. Atsu serves a “Western, educated, industrial, rich, and democratic (WEIRD)” society [12], thus replicating the health disparities he was educated to help reduce.

Were it not for their twinship and discordant migration decision, the story of these brothers would likely have a feeling of déjà vu. A conservative estimate suggests that as of 2015, roughly 13,000 physicians originating from Sub-Saharan Africa (SSA) had integrated into the licensed physician workforce of the USA [13]. While this figure did not capture the significant number of unlicensed SSA migrant physicians living in the USA, it exceeded the total number of physicians reported in 2015 by the World Health Organization (WHO) in 34 SSA countries. Such a countermovement of human resources from the world’s most under-resourced region in the presence of widespread unmet health needs requires in-depth analysis.

Current theories of international migration offer different and competing perspectives on the determinants of skilled migration [7]. *World systems* theorists, for example, read the standardization of medical curricula and practice requirements across many countries and the worldwide adoption of English as the standard medical language as a reproduction of international arrangements favoring the displacement of physicians from English-colonized countries such as Nigeria or Ghana to capital-rich countries like the USA and the UK [14]. In this macro-historical approach, the migrating doctors wield little agency in their own migration. Medical migration may better be analyzed within the global trend of resources accumulation by dispossession.

Conversely, *neoclassical economists* locate individual agency as the space of analysis of migratory behaviors. *Homo economicus*, the rational, calculating, and profit-driven individual, is the principal agent of skilled migration. His/hers is a strategic move resulting from perceived wage differentials between medical practitioners in source and receiving countries [15]. In other words, medical brain drain is likely to occur when the rate of return to medical education is perceived to be low compared to one’s skills (and social status). African physicians are likely to immigrate to the USA to maximize their permanent incomes in a measure sufficient to offset the opportunity, financial, and psychosocial costs of their emigration [16].

Meanwhile, *migrant networks* proponents claim: “Individuals do not migrate, networks do” [17]. Here, the locus of analysis is not the individual migrants, but their social networks or the sets of interpersonal relations that link individual migrants with relatives, friends, classmates, colleagues, and compatriots in source and receiving countries. Accordingly, many African physicians emigrate because other people with whom they are connected have emigrated before them and have inspired or supported them during the migration process. These migration networks become self-sustaining as prosocial immigrant behaviors are reciprocated from one cohort of immigrants to another [17, 18].

The foregoing explanations are insightful, but they also impede knowledge generation by focusing exclusively on a single level of analysis. Owing to its transnational and highly dynamic nature and its broad implications for health equity, diplomacy, development, security, human rights and social justice [19–24], the migration of health workers from resource-limited to high-income countries is an ethically-fraught, contentious, and complex problem that has proven difficult to tackle despite a universal recognition of its negative consequences and the unanimous adoption by the World Health Assembly (WHA) of a *Global Code of Practice on the International Recruitment of Health Personnel* [25].

Complexity, the “ability to negotiate among widely variant frames and stances of explanation” [26], is a defining feature of public health and social medicine, an interdisciplinary field concerned with analyzing the social structures that contribute to disease distributions and health outcomes [27]. Clarifying the relationship between various determinants of health workforce migration at different levels of analysis is a far more complex but essential approach to knowledge generation than using a one-dimensional lens. To that end, this paper describes a transdisciplinary and multilevel model suitable for the examination of complex social issues and human dynamics. Using illustrative interview data on SSA physician emigration, the paper highlights how the EPV model can be applied in qualitative research.

## Methods

### The eco-psychopolitical validity (EPV) model

**Fig. 1:**
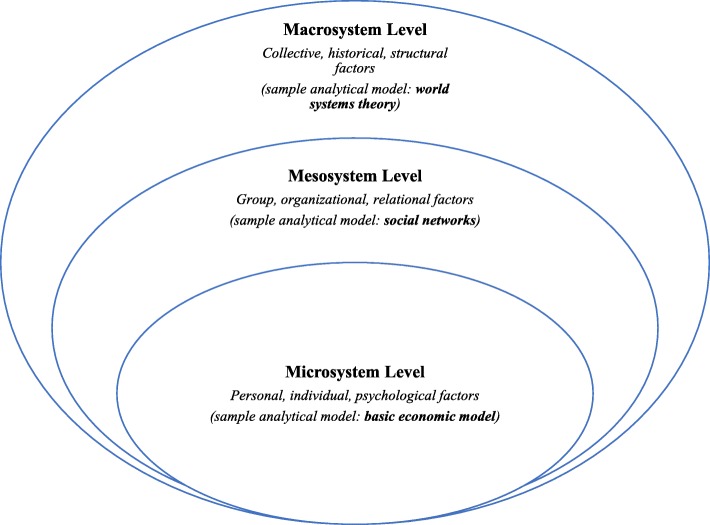
Levels of analysis of the ecological framework

**Fig. 2:**
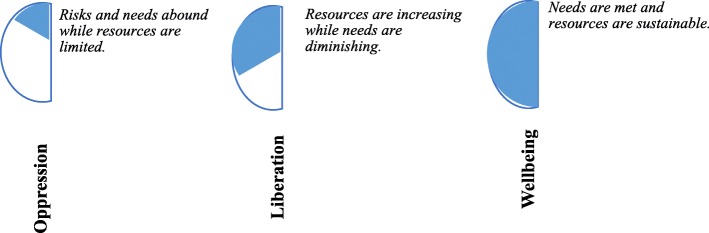
Stages of empowerment of the psychopolitical validity paradigm

The EPV model is an integrative paradigm merging ecological thinking (Fig. 1) [28] and psychopolitical validity (Fig. 2) [29]. It was conceptualized by community psychologists [30] as an attempt to examine the complexity of community problems comprehensively. Community psychology goes beyond the individual focus of the medical model of mental health diagnosis and treatment (embodied by clinical psychologists and psychiatrists) and integrates influences of a social, cultural, economic, political, environmental, and international nature to foster life-affirming outcomes at individual and systemic levels [31]. With its emphasis on social justice, advocacy, and collaboration, community psychology is fundamentally interdisciplinary and, in many ways, aligned with public health and critical medical anthropology—which views disease as biosocial, reads morbidity and mortality among the poor as the ultimate expression of structural violence [32], and defines health in terms of access and autonomy over resources needed to promote life and wellbeing [33, 34].

**Fig. 3:**
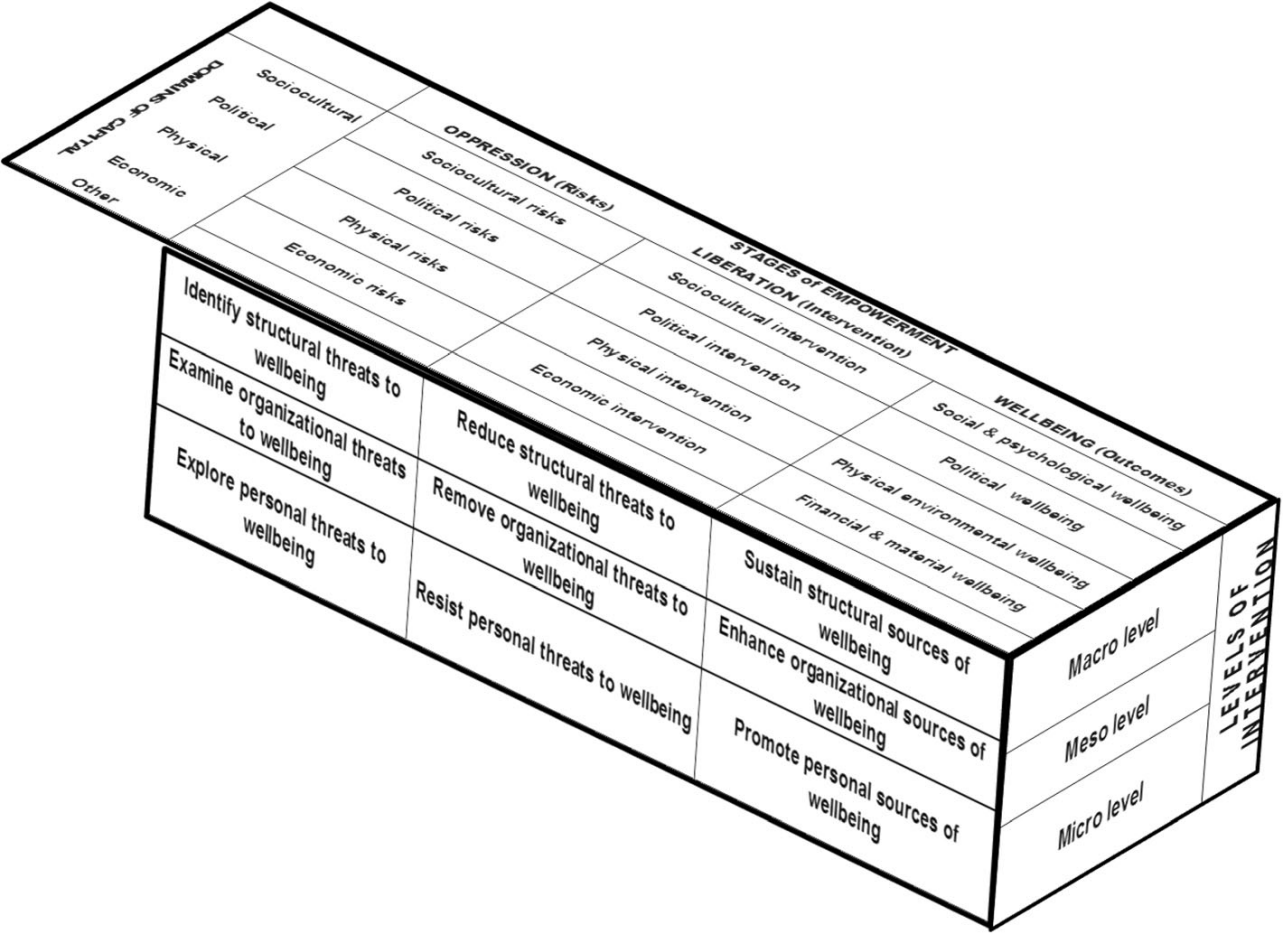
Eco-psychopolitical validity (EPV) paradigm (adapted from Christen & Perkins, 2008)

As shown in Fig. 3, the EPV model is represented as a three-dimensional structure comprising three stages of empowerment coextensive to processes of transformation or structural change along the depth, three hierarchical levels of intervention (or analysis) along the height, and four domains of capital along the width. The model is ecological because it examines human behaviors in context, that is, within the broad environment and relational systems that structure them. Ecological thinking construes migration as ‘ecological transition’ from one environment to another, and migrants as ‘ecological subjects’ nested in a vast web of complex interconnections linking near and distant actors, processes, and systems [28]. The model is psychopolitical because it acknowledges both the roles of social power and individual agency in influencing social outcomes [29]. Psychopolitical validity refers to the degree to which the analysis considers power issues and interventions promote structural change [30]. The model is transdisciplinary because it spans multiple domains of capital (or fields of expertise) and calls for a greater integration of diverse modes of inquiry in knowledge generation.

#### Levels of intervention/analysis

Variables that operate primarily at the individual or personal level are explored at the microlevel. Microsystem-level factors associated with health workforce migration may include educational aspirations, professional dissatisfaction, family obligations and reunifications, and financial self-interest, the basic building block of neoclassical economics of migration [35, 36]. The mesosystem level of the model examines both informal relational processes (e.g., socio-professional networks) and formal organizational procedures (e.g., medical school admissions, residency programs recruitment/training, and conditions of healthcare settings) that influence emigration decisions and outcomes [37–39]. *Migrant networks* theory, which focuses on relational ties between migrants and other actors in the migration channel, is integral to the mesolevel. The macrosystem level of the model explores broader structural factors identified in part by the *world systems* theory and ranging from historical antecedents to skilled migration (e.g., transatlantic slave trade, colonization, structural adjustment plans) to current national policies/regulations and transnational dynamics (e.g., US immigration policy, economic globalization) [40, 41].

#### Domains of capital

The domains of capital of the EPV model capture the diversity of factors influencing migration and the nature of intervention needed to address them. Financial incentives and other economic factors of migration are listed under the economic domain and suggest an intervention of an economic order. Political factors of migration such as freedom, democratic participation, war, and immigration policy are identified under the political domain and necessitate an intervention of a political nature. Potential cultural determinants of medical migration such as linguistic and colonial ties between source and receiving countries, westernization, and the English language are explored under the sociocultural domain. Physical factors pertaining to the built environment (e.g., social infrastructure, roads, bridges, water system, power plants) or the natural environment (e.g., topography, droughts, floods, earthquakes, extreme weather), belong to the register of the physical domain. The above four domains of capital are not exhaustive and may be expanded as necessary (e.g., legal, historical, etc.). Factors that do not fit under any of the domains may be categorized under “other.”

#### Stages of empowerment

As an analytical tool for both research and action, the EPV model contains three stages of empowerment coextensive to the process of transformation (structural change) and representing the organizing metaphors of the analysis: oppression, liberation, and wellbeing.

##### Oppression

Although brute violence such as that perpetrated in civil wars is the more visible aspect of oppression and a significant factor in mass migration and internal displacement of populations, the defining characteristic of the state of oppression is “structural violence” [42]. Structural violence refers to the social and economic conditions that determine which individuals or groups are prevented from realizing their full potential and which ones are shielded from assaults [43]. Normalized by stable institutions (e.g., justice system, school system, market) and everyday experience, the pervasive effects of structural violence seem so banal that they become nearly invisible [43]. A structural violence lens may help to explain, for example, why the average black males in the USA may have better health outcomes and a longer life expectancy when incarcerated than when living in the typical environment of non-incarcerated US black male population [44].

With respect to health workforce migration, structural violence may help to understand why nearly half of all SSA-trained physicians appearing in the AMA Masterfile by 2010 likely entered the USA between the mid 1980’s and the end of 1990’s, a 15-year timeframe coinciding with the implementation in developing countries of structural adjustment programs (SAPs) [6]. The violence of the SAPs has been documented in increased morbidity, malnutrition, excess mortality, DALYs, and harder-to-quantify destructive community processes like the large-scale emigration of skilled professionals from developing countries [45, 46]. In the same vein, structural violence may provide a lens through which to understand why the drowning in the Mediterranean of so many African emigrants and refugees seeking asylum in Western Europe has received such a tepid response from the international community that Pope Francis described it as a “globalization of indifference” [47, 48].

##### Liberation

The metaphor of liberation stems primarily from liberation theology and puts a heavy emphasis on the conditions and strivings of the poor [49]. From Erich Fromm’ binary notion of “freedom from” and “freedom to,” Prilleltensky defines liberation as the process of overcoming psychological and structural sources of oppression (freedom from) and to pursuing wellbeing (freedom to) [50]. Thus, liberation is the connective tissue and transformative dynamic linking oppression and wellbeing. Resources for liberation include the ways and means individuals and communities mobilize to free themselves from the oppressive status quo and to promote their personal and collective wellbeing. In this respect, liberation closely parallels the human development concept of *empowerment*, the process of gaining influence or control over matters of value/importance [51].

At the macrolevel, government-led policies to strengthen health systems, nationally-coordinated labor protests and coalition building among health workers unions, are examples of liberatory processes and community-level empowerment. Examples of mesolevel empowerment may include upgrade and expansion of the medical library in the local medical school, upgrade of medical equipment and working conditions in healthcare settings, and employer-sponsored in-service training to update/upgrade the skills of all staff. At the individual or microlevel, processes of personal liberation or empowerment may include continuing professional development, critical education (helpful to deconstruct and resist the dominant cultural narrative), pursuing opportunities for post-graduate specialization, and doing overtime work to generate additional revenue to support oneself and family. For the individual health workers contemplating migration, the ability to obtain a visa and move freely is a vital resource for liberation. This freedom to move appears so fundamental to human aspirations that it is enshrined in Article 13 of the Universal Declaration of Human Rights [52]. But, while theoretically migrant health workers have the right to international mobility, the nation-states they wish to enter exercise the sovereign right to control their borders and to regulate who come in (e.g., the foreign doctors) and who are left out (e.g., applicants with a communicable disease or a mental illness). 

##### Wellbeing

A transition from a condition of oppression to a status of wellbeing is the ultimate outcome of liberation—at least, that is the aspirational goal suggested by the model. It is reasonable to postulate that, by and large, health workers from the developing world migrate primarily to wealthier nations to expand their opportunity and promote their wellbeing. The World Health Organization (WHO) recognizes wellbeing as the defining characteristic of health: a “state of complete physical, mental and social wellbeing and not merely the absence of disease or infirmity” [53]. While this classic definition has not been updated since 1948, the economic, environmental, and political capitals are increasingly recognized as prominent determinants of health [54]. Thus, the metaphor of wellbeing captures subjective and objective, individual and collective sources of wellness spanning the biological, psychological, sociocultural, economic, ecological, and political domains.

### The EPV matrix

Drawing from the three-dimensional structure in Fig. 3, I developed the 36-cell matrix in Table 1 to illustrate how the EPV model can be used as a coding scheme in qualitative research. Applying a pre-established analytical framework broadly implies the use of a deductive approach to knowledge generation. The EPV model, however, is both inductive or data-driven and deductive or concept-driven. The model is data-driven because the codes generated from the thematic analysis of the interviews reflect the perspectives and experiences of respondents. The model is concept-driven because the three organizing metaphors—oppression, empowerment, and wellbeing—serve as the grouping categories for conceptually related codes. The numbers in the cells merely serve to arrange the code themes in a logical and systematic manner; they do not suggest a rank order of codes. Cells 1–12 identify threats or risk factors. Cells 13–24 catalog processes of liberation or empowerment, and cells 25–36 list wellness outcomes.

**Table 1:** Eco-Psychopolitical Validity (EPV) Model Matrix as Coding Scheme for Qualitative Data

	Oppression (risks)	Empowerment/Liberation (intervention)	Wellbeing (outcomes)
*Economic Capital*: Examines economic factors of medical migration and personnel retention			
Structural	1 Identify macroeconomic threats to countries’ health systems.	13 Identify macrolevel interventions to address economic threats to country’s health systems.	25 Identify economic policies and investments to promote long-term effectiveness and sustainability of viable health systems.
Organizational	2 Identify financial needs and organizational practices that compromise economic assets of health settings.	14 Identify diaspora-led assistance and supportive roles of non-governmental organizations in resource-limited health settings.	26 Identify economic resources and best practices for efficient management of healthcare settings.
Interpersonal	3 Identify financial needs of health workers and personnel’s behaviors that compromise economic wellbeing.	15 Identify health workers’ individual efforts to address financial needs; examine émigrés’ remittances to significant others.	27 Identify sources of health workers’ financial security and wellbeing.
*Physical Capital*: Examines physical and environmental factors of medical migration and personnel retention			
Structural	4 Identify infrastructure challenges and other threats to health systems stemming from the natural or built environments.	16 Identify national plans and actions to reduce country’s infrastructure challenges and other environmental threats to health and health systems.	28 Identify resources to sustain government’s investment in infrastructure for health systems.
Organizational	5 Assess physical needs of health facilities that compromise standards of healthcare delivery.	17 Identify health facilities’ current physical assets and maintenance efforts.	29 Identify physical resources for optimum healthcare provision and health workers’ training.
Interpersonal	6 Assess health workers’ health needs, and unmet health needs for valuable commodities, housing, and transportation.	18 Identify health workers’ efforts to meet housing and personal transportation needs.	30 Identify physical assets of health workers and family.
*Political Capital*: Examines political factors of medical migration and personnel retention			
Structural	7 Identify national and international policies and governance that threaten countries’ health systems.	19 Explore political will of country’s leadership and national health workforce planning, monitoring, and evaluation.	31 Identify national, regional, and international regulations that strengthen health systems and guarantee citizens’ rights to health.
Organizational	8 Identify organizational and bureaucratic practices that violate health workers’ rights.	20 Identify local health workers unions’ actions to improve local conditions of practice and union members’ wellbeing.	32 Identify political and organizational procedures that reinforce best practices in healthcare administration.
Interpersonal	9 Explore interpersonal dynamics that threaten health workers’ freedoms.	21 Identify health workers’ personal involvement in organized labor and political actions.	33 Identify interpersonal dynamics that promote health workers’ freedoms.
*Social Capital*: Examines social, cultural, and psychological factors of medical migration and personnel retention			
Structural	10 Identify social and cultural factors that compromise the retention of local talents and skills.	22 Identify national and regional efforts to educate, inform, and involve communities on relevant public health matters.	34 Identify social policies and cultural resources to promote the retention of country’s best and brightest talents.
Organizational	11 Identify cultural practices that undermine health service delivery and impede organizational learning.	23 Examine roles of medical schools and hometown associations in addressing shortages of health professionals and health needs in the community.	35 Explore resources to enhance team cohesiveness, effective team work, and positive group dynamics.
Interpersonal	12 Explore psychosocial factors that hinder health workers’ personal wellbeing.	24 Identify coping strategies to professional dissatisfaction and plans for upward mobility, career promotion, and family growth.	36 Explore psychological factors that increase feelings of belonging, safety, and security.
*Other*: Identifies other migration factors of significance that do not fit under any of the above domains			

### Data

The data described in this paper are presented in the form of codes and quotes. While they are essentially illustrative, they capture the gist of the main findings from a qualitative study this writer conducted as part of his doctoral dissertation research project [9]. Approved by Vanderbilt University’s institutional review board (IRB) and completed in 2012, the dissertation examined the dynamics of physician emigration from SSA to the USA. The study’s primary sample comprised a diverse group of 30 SSA-born and SSA-trained migrant physicians working in the USA at the time of the data collection. They shared their experiences of medical practice and migration through semi-structured interviews. Ethical and epistemological issues germane to qualitative data collection and analysis—namely sampling, data collection materials, treatment of participants, coding, data saturation, inter-coder reliability, respondent validation, and reflexivity—were all addressed within the parameters and limitations of the original dissertation and are not fully revisited here.

Consenting participants were selected purposively via a proprietary list from the AMA Physician Masterfile containing all SSA-trained physicians incorporated into the US licensed physician workforce and via snowball sampling which allowed for the identification of unlicensed SSA-trained migrant physicians living in the US. To present a more nuanced rendering of physician migration determinants and medical practice in SSA, I complemented the views of US-based participants by interviewing 10 SSA-based non-migrant physicians recruited among US-based participants’ classmates practicing in Nigeria, Ghana, and Ethiopia respectively. Interviews with US-based respondents were conducted in-person while those with SSA-based participants were conducted over the phone and via email exchanges. To establish reliability, a fellow health sociologist familiar with the subject matter and trained in qualitative methods volunteered as co-coder for a sample of transcripts. Minor discrepancies in code themes were identified, discussed, and a consensus was reached as to the most appropriate way to handle them.

## Results

The selected findings highlighted in this section are abstracted into three main categories reflecting the three inter-related dynamics posited as determining human migration: oppression, liberation, and wellbeing. The codes presented in Tables 2, 3, and 4 are variations of these three organizing metaphors.

**Table 2:** Examples of dynamics of oppression emerging from coding

EPV matrix cell number	Number of comments	Illustrative code (description)	Illustrative quote
1	45	*Structural adjustment programs* (Poverty-generating international economic policy)	“When I was in medical school; starting from like 1985 to 1990, that was the time when we went through a structural adjustment program, the so-called SAP, and there was a drastic reduction in the standard of living. The economy was just really, really, bad. Things looked really bleak as far as the future.” (Nigerian-trained, male, 44 years old, pathology, Tennessee, USA)
2	80	*Adverse economic impact of SAP on health settings* (Required fee for service; closure of public health facilities; meager operating budget; etc.)	“When I did my OBGYN internship in 1994, I was on-call every three days. There wasn’t a day when I didn’t lose a patient due to totally preventable causes, preeclampsia, eclampsia, or bleeding after pregnancy. The patient would come with a retained placenta. It got so bad in the operating theater that the patients had to provide their own halothane anesthetic gas before surgery. So, if they cannot afford those 20 dollars, the woman who was in labor will die. …. So, if you are practicing in an environment like that, you have to run away if you get an opportunity, because the fact is that every single day you lose a patient. And the reason why you went into this profession in the first place was to save lives. So yeah, in the late 80’s to the mid 90’s the system was totally broken.” (Nigerian-trained, male, 41, preventive medicine, Tennessee, USA)
3	14	*Adverse economic impact of SAP on health workers* (Low wages; unpaid salary; delayed pay; depleted savings; limited access to loan, etc.)	“When I left medical school, my salary was about US $40. This was 1988. I couldn’t even afford [to drive] a car if somebody gave it to me for free, because I wouldn’t have the money to buy fuel…. When people talk about frustration over lack of resources to work within the hospital, the question is: What kind of resources are you looking for when working in that environment? You do the best with what you have. Except that, your colleague who you know, was not smart enough to be a doctor, but went to wherever, and became a secretary to a bank or whatever, that colleague earns more than five times what you earn!” (Ghanaian-trained, male, 52, emergency medicine, Maryland, USA)
4	24	*Inadequate infrastructure* (Limited road network, power supply, water supply, schools, etc.)	“Hardly can you have electricity supply running for six out of twenty-four hours a day in most places. In fact, in some places they could go for a whole month without having electricity for three hours. … We even hardly remember the problem of electricity because we've come to live with it. It has become part of us, and it impacts by several magnitudes on our health systems.” (Nigerian-trained, male, 45, community health, Kano, Nigeria) “I was serving in the obstetrics ward [at Jimma Hospital], and a lady came from the countryside; her relatives brought her in, and she was pregnant. She had a ruptured uterus. … It took them two days just to come to the health clinic. They walked, because there is no public transportation, there is no road, so they had to carry her on a locally-made stretcher.” (Ethiopian-trained, male, 35, internal medicine, Washington, DC, USA)
5	80	*Substandard working conditions* (Inadequate facilities; lack of diagnostic equipment, medical supplies, drugs, running water, electricity in the workplace, etc.)	“The biggest facility where I trained, Korle-Bu Teaching Hospital, which is like the national hospital in Ghana; it is a dirty hospital. So many of its facilities are broken down; nothing is being done to fix it. The system is such that physicians are handicapped in carrying out their duties; it is like working in a jungle. … People come with chest pain, you cannot do cardiac enzymes in the night; you cannot do it on the weekends. And these are things that are time-sensitive; you can’t wait 24 hours to do some of these tests; CT scans unavailability. So, for me practicing medicine in Ghana is frustrating. A lot of physicians may be coming here [in the US] for monetary benefits and all that; it is a plus, but for me coming here makes medicine more fulfilling because I am at least able to practice medicine to the comfort level that I want.” (Ghanaian-trained, female, 35, critical care, Washington, DC, USA)
7	172	*Bad government* (Misallocation of country’s resources; mismanagement of public services; unaccountable leadership, etc.)	“My idea about Africa is that misgovernance is the biggest problem. Like, in the case of Nigeria, I know how people who are in positions of responsibility misuse resources. So, you have one person and he will take a billion dollars, 500 million dollars. This happens in Nigeria. Ok? …So, for me, even though I am a physician, the way I look at it is that the biggest impact is going to come if you can reduce corruption in Nigeria by 50%.” (Nigerian-born and trained, male, 44, pathology, Tennessee, USA) “There is a big uproar about how much politicians are earning. One of my youngest brothers is a parliamentarian in Nigeria. A typical parliamentarian makes almost close to a million US dollars every month in Nigeria. [Meanwhile], the total grant of the project that we are doing in Nigeria is not up to six months of my brother’s entitlement. Now, my brother is a representative; he is not a senator. The senator is a higher-ranking person who earns about twice what my brother earns.” (Nigerian-trained, male, 45, community health, Abuja, Nigeria) “The people that have to do something are the politicians who spend our resources. The richest people in Africa are politicians. They produce nothing, they make nothing. They’re rich. How do they get rich? They feed off the national cake.” (Ghanaian-trained, female, 35, critical care, Washington, DC, USA)
8	21	*Corruption in public health settings* (Systemic culture of corruption in government-led healthcare organizations)	“It is such an intricate web. If a contract is being given, the person in the ministry gets a cut, the auditor gets a cut. The contractor inflates the rates. The person who is in-charge of accounts at the ministry and the hospital itself gets a cut. The storekeeper gets a cut.” (Nigerian-trained, male, 41, preventive medicine, Tennessee, USA)
9	21	*Cynicism about political leadership* (Dystopian view of own native country and Africa; distrust of African politicians; civic disengagement, etc.)	“When I was in the UK, there was this Nigerian lawyer who I went to talk to, and then when I told him I was going back to Ghana, he asked: ‘Why do I want to go back? There is no point coming to Africa, there is barely any good in Africa, you better stay in the UK’.” (Ghanaian-trained, male, 48, internal medicine, Accra, Ghana)
10	91	Insecurity (Widespread armed robbery; kidnapping; inadequate policing, etc.)	“What's happening in Nigeria, it's like, despite the democracy and the elections, there is too much insecurity for yourself and your family to kind of take that chance [of returning] no matter how much they may be willing to pay you. So now, the push factor [of emigration] may not be money, it will be more of security and technology.” (Nigerian-trained, male, 45, community health, Abuja, Nigeria)
11	18	*Cultural threats to healthcare* (Illiteracy; reliance on traditional healers; hospital visit as last resort; stigma, etc.)	“From my little experience working in Ghana, a lot of times, people come late to the hospital.” (Ghanaian-trained, female, 35, critical care, Washington, DC, USA) “Many patronize quacks, and the so-called traditional healers who deceive the unsuspecting general public.” (Nigerian-trained, male, 45, community health, Abuja, Nigeria)
12	149	*Loneliness abroad* (Feeling of estrangement; limited support network; longing for home, etc.)	“Here is very lonely. You don't have people to talk to; that's my biggest problem. I miss that social aspect a whole lot. I need to go back home and reignite that because here it's almost gone.” (Nigerian-trained, male, 45, critical care, Tennessee, USA) “Coming here you realize that money cannot give you happiness. … Social support is a big issue in America. But, back home there is this support system that I think has a positive impact on people.” (Nigerian-trained, male, 33, internal medicine, Washington, DC, USA)

**Table 3:** Examples of empowerment dynamics emerging from coding

EPV matrix cell number	Number of comments	Illustrative codes (description)	Illustrative quotes
13	33	*Private health sector investments* (Expanding private health sector; private health insurance scheme, etc.)	“Another area which is also gradually expanding when it comes to medicine, there are lot of private universities now setting up medical schools. And there are lots of private hospitals also coming up. And you may have a lot of doctors now weighing options as whether to work in the government sector or to go purely private. And we have a lot of those who are in private sector and they are happy.” (Ghanaian-trained, male, 48, internal medicine, Accra, Ghana)
15	84	*Remittances* (financial support of family and significant others in home country, etc.)	“When I was in Ethiopia, I don't think I have given any substantial amount of money to my family, even occasionally. I don't remember giving them any helpful amount of money. But, since I came to the United States, I have sent I think a good amount of money to my family, at least to make them have no problem with their day-to-day basic lives.” (Ethiopian-born and trained, male, 38, internal medicine, Washington, DC, USA)
17	31	*Physical assets of private health settings* (Optimization of facilities, equipment, and human resources in private health settings, etc.)	“I think in the private health sector, things are better because they are there purely to make profit, so they take better care of their equipment. They are charging patients. So, they are able to maintain things. And also, people's expectations of the private hospitals are higher, so they need to live up to expectations. Because if you own a private hospital and people are not satisfied with your service, then they can go to another hospital. So I think competition and all that has helped make them better. … But, I always say that private hospitals are usually run by the same doctors who work in the public hospitals. They're the same people, but in a different setting. And their performance varies. Because, when they need stuff at the private hospital, they get it. They need it in a public hospital, it will take, what, two, three, four months?” (Ghanaian-trained, female, 33, public health, Accra, Ghana) “The beneficiaries of public institutions and government policy seem to have deliberately killed public institutions to allow the private ones to flourish. …It is like robbing Peter to pay Paul.” (Nigerian-trained, male, 45, community health, Abuja, Nigeria)
19	17	*National labor unions* (Establishing health unions; mobilizing health workers nationally; organizing strikes; collective bargaining, etc.)	“Prior to our action, what the doctors were receiving in terms of total emolument was really, really, miserable; especially those doctors who were working exclusively in the academic setting as lecturers. … One of the good things that we succeeded in achieving from the strike was to make locum tenens legal, once the doctors finish their regular clinical work. Prior to that, locum tenens was illegal.” (Nigerian-trained, male, 45, community health, Abuja, Nigeria)
20	25	*Visa network and channels* (Network of actors and organizations in the visa-granting process; manner of entry in the US)	“It was my penultimate year. I was one of the recipients of the Green Card Lottery. So, I think that played a huge role in the certainty for me to immigrate to the United States.” (Nigerian-trained, male, 41, psychiatry, Tennessee, USA) “I was admitted to the University of Boston in 2007 for the MPH but could not afford it. I was again admitted to University of Arizona in 2008 for the MPH but could not afford it. I wanted to use all these as spring boards for my emigration plans.” (Nigerian-trained, male, 45, community health, Abuja, Nigeria)
21	30	*Defining own identity* (Resisting complete assimilation in host country; accepting or declining host country citizenship; asserting one’s national origin, etc.)	“When I meet colleagues, friends, or just strangers, and we talk; they ask me: ‘Where are you from?’ What they mean is: ‘You have an accent’ or ‘I could detect, I could feel out that you weren’t born and raised in the U.S. You are from somewhere else.’ So, as long as people here are asking me where I am from, and are expecting that I answer, ‘I am from this country, or that country,’ then, I will never be able to claim complete identity here [in the US].” (Nigerian-trained, male, 44, pathology, Tennessee, USA) “I am Ghanaian. The passport you hold doesn’t say where you come from. Where you come from is where you feel you come from in your heart. And, I am Ghanaian. So, when we come to the point where you can hold a Ghanaian passport and go anywhere without the limitations for visas and stereotyping and all that then probably one would be comfortable holding a Ghanaian passport.” (Ghanaian-trained, female, 35, critical care, Washington, DC, USA)
22	39	*Regional and national residency training institutions* (West African College of Physicians; West African College of Surgeons; Ghana College of Physicians, etc.)	“Apart from the West African College of Surgeons, there was a new Ghana College of Physicians and Surgeons that started training surgeons in the country at that time. So, I decided to join.” (Ghanaian-trained, male, 38, obstetrics and gynecology, Accra, Ghana)
23	21	*Diaspora-driven support of home-based health organizations* (Medical trips to the home country; donation of medical supplies to health organizations in origin country, etc.)	“At the individual level, we all do what we can, but we need a collective decision, a collective effort to make a significant change. Some of us, including myself, gather resources at our own expenses and take them home. The local village where I came from, I spent at least $5000 in the last three/four years, helping them. I gather the medications and I appeal to other people, and I take various medications there. But the question is: Do they end up in taking care of the people? The staff will take the medications. For all I know they might sell it or do something else with that. That is not the appropriate way of helping the people. There is no comprehensive way to do that. So, at the individual level you help various people. But, at the collective level, that is a different area altogether. Some individuals within the Ghana College of Physicians and Surgeons, the means by which you got me, wanted to establish a parallel organization, meaning building a hospital in Ghana that, we had hoped would practice in the same way we do in the US and hopefully become a focus to bring some change. And, that didn’t work out so well after various people invested various amount of money. The guy who forwarded your email to me was one of the people. He spent about $35,000. I only put in $10,000. It is now zero. So, you know, we have hopes, and then we have realities that occur along the way.” (Ghanaian-trained, male, 52, emergency medicine, Maryland, USA)
24	172	*Personal and professional development* (Advanced training and specialization; peer-reviewed publications; international exposure and recognition; achievement drive, etc.)	“At the onset when I left, I had believed that when I am done with all these specializations I want to go back to Cameroon. I want to be able to set up practices and implement things which are actually not there, or may not be readily available, especially in the fields I chose. So, I came to the U.S., after been to England and other places; I came to the U.S. and did a residency at Henry Ford Hospital in pediatrics and adolescent medicine. And after residency I worked for Wayne State University Hospital, the Detroit Medical Center, as a physician. But then again, I felt like I needed to be a fellow. And I needed to have more studies. So, at the end of a three-year period, I took up a fellowship in adult and pediatric allergy and immune system disorders at the Louisiana State University Health Science Centers, where I finished and currently is board certified in the fields of pediatrics, in the fields of adult and pediatric allergy and immune system disorders.” (Cameroon-born, Nigerian-trained, female, 44, pediatrics, Michigan, USA) “I was in the U.S. for three months as a clinical observer. I've gone to different countries attending seminars, workshops and conferences. And I’ve published in the international journals. When I was in the U.S. actually, I stayed in Baltimore in the St. Agnes Hospital. And, I remember the program director of the residency training at that time asked me if I was interested to come back to the U.S. He asked me to go and do the USMLE exams and he offered, if he is still the director, to give me a place. But, I actually was not interested. I really wanted to go to the U.S. to get exposed to training, and not because of lack of opportunity.” (Nigerian-trained, male, 41, cardiology, Kano, Nigeria)

**Table 4:** Examples of sources of wellbeing emerging from coding

EPV matrix cell number	Number of comments	Illustrative codes (description)	Illustrative quotes
25	37	*Expanded opportunity in the health labor market* (Relative abundance and fairness in distribution of employment opportunities)	“Back home, there is a ceiling. Once you achieve certain specialty you can't move further in your career development. But, in the US, the sky is the limit, I would say. So, you have a lot of opportunities to go ahead in your career.” (Ethiopian-trained, male, 35, internal medicine, Washington, DC, USA)
27	50	*Financial comfort* (Competitive income; timely remuneration; financial security; personal savings, etc.)	“I am getting paid well for doing something that I like. And, I am paid in such a way that I can afford to help people around me also get a better education. All my brothers and sisters who are younger than me have been able to leave Cameroon because I have been practicing medicine in the US.” (Cameroonian-trained, male, 43, pediatrics, Virginia, USA) “I consider myself among those that are a bit on the better side when it comes to standard of living. I earn quite a lot of money according to my standards, because it is enough to take care of a lot of my problems and that of my family.” (Nigerian-trained, male, 45, community medicine, Kano, Nigeria)
29	32	*Attractive work environment* (World-class facilities and equipment; state-of-the-art technology, etc.)	“When I went to George Washington University Hospital while I was there in the US, the place was clean and sweet. I mean, it was nice to work in there, you see? But then back home, the environment is not checked well, the place is not maintained.” (Ghanaian-trained, female, 33, public health, Accra, Ghana) “Here [in the US] you see state-of-the-art technology, investigations, lab tests. You don't hear about those problems you hear of in Ethiopia regarding paying for prescription, paying for lab tests, paying for imaging studies. And sometimes, even the people you treat don't see their bill. It's the insurance companies who pay.” (Ethiopian-trained, male, 38, internal medicine, Washington, DC, USA)
33	3	*First-class citizen status* (Right to dignity and equality; freedom from discrimination; loyalty to home country, etc.)	“Well, I am staying in my country, because I love Ghana, and I love being a first-class citizen in Ghana. And, I believe that no matter what you do, where you go, and who you become, forever, you'll never be a first-class citizen in anybody else's country but your own.” (Ghanaian-trained, female, 33, public health, Accra, Ghana)
35	14	*Institutional support* (Immigrant-friendly and culturally sensitive residency programs)	“I like everything about my residency training program. I like the job satisfaction. I like the fact that you see results. I haven’t had any experience with racism because of course I am at Howard University. So, I like it. It is an extension of Ghana to me.” (Ghanaian-born and trained, female, 35, critical care, Washington, DC, USA)
36	19	*Securely attached to home* (Attachment to homeland; sense of community; feeling of belonging; place identity, etc.)	“I have never regretted practicing in Nigeria, and it has never crossed my mind to go and practice outside Nigeria. …I would like my children to have a sense of belonging. Yes, I feel they have their pride when they grow up in their own country and believe that they're at home.” (Nigerian-born and trained, male, 45, community medicine, Maiduguri, Nigeria) “We believe that Ghana is our home, and we stay in Ghana, and we work in Ghana. And, then if you need to go for a holiday or travel, you save up some money and then you go. But, by all means you come back to our home, and we will be here.” (Ghanaian-trained, female, 33, public health, Accra, Ghana)

### Sources of oppression

Two potential research questions that could be addressed by the exploration of sources of oppression are: What are the professional and personal needs of SSA migrant physicians? What structural and interpersonal factors are likely to intensify these needs? A close examination of Table 2 suggests that a host of factors within and beyond the country health systems compromise SSA health workers’ wellbeing and influence their decision to emigrate.

#### Economic crisis and poverty-generating policy

Senior and mid-career physicians who experienced the socioeconomic impact of the structural adjustment programs (SAPs) in the mid 1980’s and throughout the 1990’s lived through currency devaluation, inflation, spike in commodity prices, and widespread poverty resulting from the implementation of the SAPs. They painted a grim picture of life in their home countries with terrible memories of material deprivation (Cells 1, 2, 3). With a monthly salary of $40, their economic circumstances were dismal and that of their patients even grimmer. As one respondent explained, “it got so bad in the operating theaters that the patients had to provide their own halothane anesthetic gas before surgery. So, if they cannot afford that $20, the woman who was in labor would die.”

#### Substandard conditions of service and limited infrastructure

While acknowledging a relative improvement of physicians’ remuneration in recent years, junior physicians, from Ghana to Ethiopia, deplore the substandard working conditions in government-run hospitals (Cell 5). The challenges arising from the lack of diagnostic equipment, drugs, beds, disinfectants, gloves, and detergents in healthcare facilities are further compounded by the shortage of basic social infrastructure such as water, electricity, and roads (Cell 4). Nigeria, the largest economy in Africa, is crippled by epileptic power supply. In health facilities where the necessary medical equipment is available, often, there is no electricity to run it. Ghana, one of the most stable democracies and fastest growing economies in Africa for several years running has fewer challenges with electricity, but a critical shortage of clean water. A Maryland-based senior emergency physician contemplating retirement in his native Ghana in “the next 8-10 years” reflected:

*“What I see as the basic needs of individuals is water, running water. It is worse than it was 50 years ago, even in the capital. I mean, that is a basic problem that cannot be solved by individual doctors. When a society doesn’t think that basic running water is a fundamental need of a developing nation, whatever you do in terms of treating diseases that occur as a result, it’s not gonna help*. … *My wife and I are going to go to Ghana to retire. But, our hope was that we give the children to buy into that idea. So that, when they come home they would be happy. A gated community for which we paid US$68,000, by Ghanaian standards that’s a lot of money; a beautiful place. But, guess what? Water flows once every two weeks. One day out of two weeks! …Over the last 15 to 20 years governments have come and go, but this fresh water lake [Lake Volta] has been there for 50 years, ok? What is preventing people to think about [using] it? Is that any less important than organizing the African Cup of Nations? Think about that. I mean, for 50 years, we don’t have water. And, when they talk about Ghana in the media, they say, ‘Oh Ghana is improving’. If this is the best that we can offer, then what about the rest of Africa?”*

An Addis Ababa-based public health physician explained that, despite an immense need for hospital-based quality care and a limited number of tertiary healthcare facilities in Ethiopia, “hospital utilization rate is only 30%.” On the one hand, this very low utilization rate is due to “geographic inaccessibility” of many health facilities, inadequate road networks and limited public transportation. On the other hand, in rural areas especially, it is a consequence of financial constraint and a reliance on traditional healers as a first port of call for the sick and an affordable alternative to hospital-based care (Cell 11).

#### Bad government and public corruption

Investment in social infrastructure is primarily the responsibility of the government. Reflecting on the adverse impact of bad government on health systems and health workers’ wellbeing (Cells 7–9), respondents unanimously decried by the lack of political will by politicians who “feed off the national cake.” The rhetorical question by a US-based Cameroonian respondent echoed this general sentiment: “Why are all our dignitaries in government going abroad to get their care done as opposed to beefing up the country health systems?”

The perverse effects of bad governance on the health system are far-reaching. At the mesolevel, the mismanagement and misappropriation of the country’s scarce resources makes the healthcare procurement process highly vulnerable to collusion and corruption. As noted by a Nigerian-trained émigré, “if a contract is being given, the person in the ministry gets a cut, the auditor gets a cut. The contractor inflates the rates. The person who is in-charge of accounts at the ministry and the hospital itself gets a cut. The storekeeper gets a cut.”

Furthermore, bad governance compromises the day-to-day management of public health settings. “As politicians decide the staff to be employed,” administrators of government-run hospitals may not necessarily be the most competent, but the most connected. At the micro level, perceptions of endemic corruption and unaccountability of the political elite can inure physician-citizens to such practices, rendering them highly cynical about their government and leading to a dystopian view of one’s own country. Under such a scenario, migration is seen as a necessity and return as a hazard (Cell 9). Another important deterrent to permanent return is the elevated security risks that prevails in many source countries. While I coded it under the social domain (Cell 10), the main drivers of insecurity in Nigeria are political, stemming from religious extremism in northeastern Nigeria, and local resistance to “environmental degradation” from petroleum pollution in the Niger Delta.

### Processes of empowerment

Under the rubric of empowerment or liberation, research questions that can be answered include: What personal resources and opportunity structures are available to SSA physicians for implementing or halting their migration plans? Once they have settled in the USA, what are their long-term plans vis-à-vis returning or supporting their native countries? How successful are they in carrying out these plans?

Table 3 highlights some significant dynamics, most relevantly the importance of professional development as both a migration driver and a means for personal empowerment (Cell 24), the channels used by the émigrés to obtain a visa (Cell 20), the increasing role of the private health sector (Cells 13 and 17), the empowering role of health worker unions (Cell 19), émigrés’ ongoing negotiating of their identities (Cell 21), and the medical diaspora’s support of the native country (Cells 15 and 23).

#### Professional and personal development

If education is a tool for personal development and liberation, post-graduate medical education empowers migrant doctors to meet their ultimate career goals in the USA. The Bachelor of Medicine, Bachelor of Surgery (MBBS) degree obtained in the home country facilitates their professional integration into US Graduate Medical Education (GME) residency programs and enables their transition from a domestic work environment often characterized by “very rustic” medical facilities to US teaching hospitals endowed with “state-of-the-art technology, investigations, and lab tests.” Although limited, opportunities for post-graduate medical training are available in the SSA countries surveyed. Nigeria and Ghana-based respondents were all fellows of the West African College of Physicians (WACP) or the West African College of Surgeons (WACS), the largest post-graduate medical programs in West Africa (Cell 22). Further, several SSA-based respondents reported staying current with the latest developments in their field of specialization by participating in international seminars and conferences and by collaborating with their US-based colleagues. Hence, the possibility for international consultancy and collaboration and the availability of residency training opportunities in-country has diminished the need for emigration for many West African medical graduates.

#### Visa channels and networks

To “make it in the United States,” migrant physicians first need to make it to the United States. This requires obtaining an entry visa. US-based informants obtained their US visas through four main avenues. The preponderant route of entry was skill-based and entailed the support of a US academic institution or a US residency program to obtain either a F-1 visa (international students), a J-1 visa (exchange scholars), or a H-1B visa (foreign professionals with special skills). The second most popular entry channel was through the “Visitor visa.” With an ulterior motive of pursuing long-term medical practice opportunities in the USA, holders of this short-term visa officially entered the USA as tourists (B-1 visa) or for business purposes such as attendance of scientific conferences (B-2 visa). One respondent who used this visa channel explained that he entered the USA in 1994 as a FIFA World Cup attendee. He eventually adjusted his immigration status and prepared for examinations for admission into GME residency while working as a doorman in a hotel in Washington DC. Five respondents came to the USA as spouses or fiancés of US citizens (K-1/K-3 visas). Two others won the US State Department-sponsored Diversity Lottery visa within a year of graduating from medical school and immigrated to the USA as permanent residents. Not all visa applicants are successful. One Nigerian-based informant who described himself as “totally dissatisfied with the quality of life and professional fulfillment” explained his unsuccessful attempt to get a visa to North America:

*“I started the Canadian immigration application last year but stopped it because I need a minimum of 60,000.00 Canadian dollars in my account in addition to the cost of planning and funding of my travels and that of my family members before my application could be considered. I was admitted to the University of Boston in 2007 for the MPH but could not afford it. I was again admitted to University of Arizona in 2008 for the MPH but could not afford it. I wanted to use all these as springboards for my emigration plans.”*

#### Growing private health sector

Reportedly, the private sector of healthcare is expanding rapidly in Nigeria and Ghana, creating more professional opportunities for the medical graduates to practice locally, more competition for skills in the national health labor market, and more choices for patients with the ability to defray the cost of their care. While the growth of a competitive private health sector may represent a positive development for a country’s health system, such development at the expenses of a weak public health system is concerning. A Nigeria-based doctor who works with a Nigerian health NGO and volunteers as a medical faculty in a public university indicated that private practice is “completely unregulated” in Nigeria and too often, the owners of private practices are public employees who take advantage of their positions in the government to embezzle resources needed to build their private clinics. “The beneficiaries of public institutions and government policy seem to have deliberately killed public institutions to allow the private ones to flourish. … It is like robbing Peter to pay Paul,” he lamented.

#### Labor unions’ action

About two decades earlier, the above informant was the national secretary of Nigeria’s National Association of Resident Doctors. Through collective bargaining and strikes, they successfully compelled the then Nigerian military government to increase physicians’ salaries and to allow them to work in private practice after finishing their daily duties in public hospitals. Prior to their successful mobilization, doing locum tenens or holding a private practice was against the law for Nigerian physicians employed in the public sector. Similarly, Ghana-based informants reported that their economic circumstances have improved “markedly” thanks to medical doctors’ “discord and industrial action” conducted over the years by the Ghana Medical Association. However, this has come at a cost as government-led “doctor bashing” has diminished the high regard doctors used to enjoy among the population. “Previously, when doctors went on strike for salaries, the whole nation backed them,” an informant explained. “But now, people get upset. People think that a lot has been done for doctors. But, that’s also because the politicians have made it like that.”

#### Identity negotiation

While expanding the professional and economic opportunities of émigrés, migration also entails an ongoing process of identity negotiation which involves asserting how they see and define themselves and challenging how others see them. Identity enables the émigrés to claim membership in social groups with shared experiences. Individuals’ identification with a given group confers them with the symbolic capital invested in the group and makes them more amenable to promote/defend the interests of the latter. Judging by several respondents’ statements, many SSA migrant physicians do not want to compromise their national origin: “I am Ghanaian to the core.” “I am a proud Nigerian.” “That one is non-negotiable.” “I do not see myself as an American.” The authors of these soundbites are naturalized US citizens. Yet, they defined themselves almost exclusively in terms of the cultural referents of their native countries.

Other respondents, however, conveyed an attachment to both their native and host countries and could not wholly embrace or reject either. A feeling of belonging to “this country, and that country” appears to be the transcendental feature of their hyphenated social identities. This sense of double consciousness is compounded by a pervasive sense of alienation as captured by the following quote:

*“When I meet colleagues, friends, or just strangers, and we talk; they ask me: Where are you from? What they mean is, ‘You have an accent or, I could detect, I could feel out that you weren’t born and raised in the U.S. You are from somewhere else.’ So as long as people here are asking me where I am from, and are expecting that I answer, “I am from this country, or that country”, then, I will never be able to claim complete identity here.”*

The Nigerian medical graduate who authored the sentiment above has been living in the USA for over 25 years, becoming a US citizen in 2005, achieving full professorship in pathology and urological research at an elite US university a few years later, and receiving induction into the prominent American Society for Clinical Investigation subsequently. However, despite these stellar accomplishments, fully embracing the American national identity is an aspiration beyond his control. The sense of double consciousness reflected in the accounts of émigrés is associated with an enduring longing for home. Many reported missing their native “food” and “culture”. Despite the relative ease of “picking up the phone and calling”, they miss being around family and friends, and sitting down at sunset and chatting with significant others after the “Maghrib prayer.” To allay their longing for home and the lingering separation anxiety associated with migration, some émigrés return home socially through involvement in hometown associations, diasporic organizations, alumni groups, and humanitarian missions in the home country.

#### Diaspora’s resources

The extent to which African-based medical organizations such as the Ghana Medical Association and the Ghana College of Physicians and Surgeons leverage the resources available within the medical diasporas to improve healthcare and promote health in the home countries was not adequately examined. However, the unflattering experiences reported by some émigrés who have attempted to lend support to health organizations in the home countries suggest that the resources of the medical diasporas are not exploited sufficiently by the actors located in the home countries. Commenting on physicians’ obligations vis-à-vis their country, a Ghanaian émigré practicing internal medicine in the Washington, DC metropolitan area reflected:

*“You know, yeah, definitely you have feelings of obligation to help in whatever way you’d be allowed to help, let me state that. Some of this is even financial. …But, the big thing is that, as much as you are trying to give, there is no hand extended to receive. And, I speak not only for myself but for many physicians like me who want to give back one way or another, who are happy to teach in the medical school, do clinic stuff like that. But, the number of obstacles you have to overcome just to do that makes it ridiculous. So, anybody who is doing that, is doing it really after overcoming a lot of hurdles and obstacles. And, of course if you have to go through all that, then the potential impact would be less than it could have been.”*

Echoing a similar sentiment, the Maryland-based past president of the now defunct Cameroon Physicians Association, commented:

*“If the institutions at home will take it as a priority and organize resources that are coming from abroad, it will help. … If the country will say, give incentives to physicians in the diaspora to participate in a project that they create, I bet you that funds will flow directly to it like nothing. But, if people have to take individual initiatives, then you have to have people who are really entrepreneurial like Dr. CC to follow that kind of course. …I have had to deal with the actors in Cameroon directly. And, I found out that most of them are not interested in the big picture. They are mostly interested in ‘what is in there for me?’ And so, that is what my experience has been. …You have a lot of resources that are in this country that we need to use. Like for example, when Bush was President, he had an AIDS for Africa program, where countries would apply to get funds to help HIV orphans and offspring of HIV positive patients. And, we tried to tap into that fund. …The demand had to come from the home country, and the funds had to be sent directly to the home country. So, what we decided to do was to call the person who was in-charge of HIV disease at home and explained that, ‘we know how to write the grant application. We will write the grant application, send it to you. You sign it, send it to them, and they send the money to you.’ They were not interested. So, with all that kind of experience, you begin to ask yourself that, what else can you really do? So, they have to be motivated on their own end.”*

By all accounts, the resources sent home by the medical diasporas are not properly pooled. Most of these resources are provided on an individual-basis and are primarily directed to family members in the form of remittances. Many émigrés reported sending monies home to significant others sporadically. Most declined to provide any specifics on amount. A Nigerian-trained medical graduate and resident in internal medicine at Howard University Hospital observed with a mixture of humility and pride: “That one is personal, but just know that there’s nobody I know now in Naija that can die because of $1,000.” Respondents who disclosed specific figures reported varying amounts of money ranging from “about $100 a month” to “maybe $5000” a year. The monies are sent home for various reasons including school fees for children and to support émigrés’ aging parents.

The entrepreneurial doctor cited as an example in the above quote (Dr. CC) has been empowering the health sector of his native Cameroon through the small NGO he created two decades ago. Up to four times a year, he travels to Cameroon with essential medications and medical equipment to address unmet health needs in his village and in the urban neighborhood of Yaoundé, the Cameroon’s capital in which he grew up. His involvement stems from the feeling of moral obligation he has toward his country.

“*I feel indebted to the Cameroonian people. From elementary education, to secondary education, to medical school, it was all free for me. So, in some ways the Cameroon Government contributed in making me a physician and I have always felt indebted to them, and I have always expressed it to the authority that be, and I have always worked, as much as I can, in making healthcare more accessible to Cameroonians. To that effect, once I got into residency in the USA, I created a nonprofit organization called GSC. We provide free medications and medical supplies to the Cameroon people. Just last year in 2010 we refurbished two hospitals in Cameroon, one in Bota, Limbe, and another one in Banguem. … I have a medical mission that goes to Cameroon at least twice a year. Last year I went like four times; it depends on the resources that I have, but at least I go twice a year with medications and medical supplies. …I usually go with between 22 to 30 suitcases of medication and medical supplies. I take nebulizer machines, blood pressure machines, EKG machines, urinalysis machines, and then I do my tests on the patients that I examine and then give them the medications. In Yaoundé, where my parents are, every morning from 8 am to 2 pm I see patients. There is a clinic called HS Clinic which also provides quite a lot of free consultation. So, I put part of my medication in HS Clinic. I take some of the medications and equipment to the village which is in Bagangté. And then I have a medical supplies box which is under my dad’s foundation for those who cannot pay for the medications. When they get the prescription script, they just go to the box and get the medication.”*

### Sources of wellbeing

Exploring sources of wellbeing may help to provide some answers to the following research questions: How satisfied are migrant (and non-migrant) SSA doctors with their life and career goals? What factors influence these levels of satisfaction?

As suggested by Table 4, both objective and subjective factors determine SSA physicians’ wellbeing. These factors include financial security (Cell 27), opportunity for professional growth, choice, and upward mobility afforded by the large and competitive US health labor market (Cell 25), supportive work environment (Cells 29, 35), and sense of belonging and place-attachment (Cells 33, 36).

“*The compensation here, as a physician, is substantially different from Ghana, regardless of how much taxes you pay. Now they are paying physicians well in Ghana; by Ghanaian standards, they are comfortable. But, absolutely, the kind of salary you get here as a physician, anywhere from $200,000 to $600,000 a year depending on what you do, is huge. And it is for doing what you like. So, for me, it is a big draw.*”

#### Financial security

As reflected in the quote above, the expectation of a high and steady salary after post-graduate residency is a significant pull factor of migration for many SSA medical graduates plying their skills in the USA. While often, their “energy is used up” for working extensive hours, this expense of “energy converts into money” at the end of the pay period and provides them with a financial security that is hard to achieve in their home countries and in most professions in the USA. Owing to the inclusion of several resident (unlicensed) physicians in my sample, the figures reported by US-based respondents were not as high as suggested above but range from US$120,000 to over US$400,000 for licensed physicians. The lone participant who reported earning US$120,000 annually worked 40 h/week, that is, at least 10 h/week less than most of her peers. At the time, her salary was more than twice the median annual income for US households (about US$50,000). Hence, by most standards, she was comfortable. “I am really happy to be a medical doctor, and if I had to start all over again, I would still be a medical doctor,” she stated.

#### Enabling environment

Professional satisfaction is also determined by the enabling environment. At the mesolevel, well-resourced, well-designed, and well-maintained teaching hospitals and non-academic healthcare settings make healthcare practice more attractive and fulfilling for the migrant doctors. At the macrolevel, operating in the world’s single largest health labor market which is the USA provides the migrant doctors with more choices and opportunities for upward mobility. Once they have completed residency, they can pursue licensing and establish themselves in any of the 50 US states and in the District of Columbia. High achievers can to seek further specialization and sub-specialization. Further, the advanced infrastructure of the USA makes life more convenient and emergency response more efficient. One Tennessee-based informant described the convenience of practicing in the USA as follows:

*“I treated one woman who had a bleeding brain. I called a neurosurgeon. I called a special institution. They came here, flew the patient, did almost eight hours of surgery on the patient and she’s back to work now. She’s back on the floor working! I mean where else can we do stuff like that?”*

#### Attachment to home

While no doubt, economic resources are the basis of material wellbeing, socio-cultural and interpersonal factors appear equally important for the wellbeing of many physicians, especially in the home country. A Nigeria-based respondent who acknowledged that “all is not rosy” in Nigeria expressed a deep sense of gratitude for staying home and tending to his late mother and for continuing to “take good care of his wheelchair stuck father”, an obligation he would not be able to take on had he migrated. With a mix of national pride and contentment, an early-career public health physician highlighted the significance of her social support network in Ghana and the intrinsic reward she derives from practicing in her native country:

*“Well, I am staying in my country, because the truth is that I love Ghana, and I love being a first-class citizen in Ghana. And, I believe that no matter what you do, where you go, and who you become forever, you’ll never be a first-class citizen in anybody else’s country but your own. In Ghana, when I open my mouth, wherever I work, wherever I go, this is my home. …I can’t be in the US and the only thing I have is my husband or my children. I mean, there’s a bigger family. And, there’s a bigger circle of friends. And, I mean, I have really good friends who think like I do. We believe that Ghana is our home, and we stay in Ghana, and we work in Ghana. And, then if you need to go for a holiday or travel, you save up some money and then you go. But, by all means we come back to our home, and we will be here.”*

## Discussion

The social determinants of health and health inequities are complex, multidimensional, and dynamics, and “epidemiologically disentangling and isolating [their] multiple influences on a nation’s health” is daunting [46]. Ten years ago, the Commission on Social Determinants of Health (CSDH) stated that health inequities stem from the inequitable distribution of power (political capital), money (financial capital), and resources (e.g., physical, human, social, and symbolic capitals), and tackling these structural drivers at the local, national, and global levels should be a priority. Among its principles of action, the CSDH recommended to “measure and understand the problem and assess impact of action” [55].

Measuring and understanding social determinants adequately requires an ability to integrate explanatory frames and complementary methods from diverse fields of scholarship [26]. This paper sought to contribute to such understanding by describing a transdisciplinary and multilevel model for research and action, thus far insufficiently tapped, but with the potential to systematize the exploration of social determinants of health and inequities. Using health workforce migration as an example of a complex social issue with health inequity implications, this paper articulated how the three organizing metaphors of the proposed EPV model namely oppression, empowerment, and wellbeing structure migration and can be examined systematically by exploring both individual and contextual factors.

The EPV model suggests that physicians migrate to promote their wellbeing. The wellbeing of physicians is essential for effective patient care and improved healthcare system performance. In the USA, the favorite destination of migrating physicians, high rates of depression, burnout, and increased suicide risk are commonly reported among physicians [56]. The ever-growing recognition of these occupational risks has led to the recent publication of a *Charter on Physician Wellbeing*, which outlines guiding principles and key commitments that can help governing bodies and policy makers develop policies aligned with best practices that promote physician wellbeing. At the meso-organizational level, the charter can inspire medical organizations to adopt strategic priorities and interventions that maximize meaning, engagement, and job satisfaction among healthcare team members [57]. The charter has been endorsed by major medical organizations including the American Medical Association, the American Psychiatric Association, the Association of American Medical Colleges, and the Institute of Healthcare Improvement [58].

Whether physicians leaving Africa to practice say in North America can fully attain wellbeing abroad when their emigrations compromise the health and life outcomes of fellow compatriots in the native countries is debatable. In the face of anti-immigrant sentiments and rising nationalism in many Western nations, the pursuit of wellbeing by way of emigration may become even more problematic as migrating doctors fleeing structural violence at home may encounter fiercer resistance and even outright discrimination abroad [59, 60]. Against this backdrop, “brain waste,” the deskilling of highly skilled migrants due to prolonged unemployment or underemployment in the host country may become the experience of many migrant physicians [61]. With limited social capital in the host country, the realization that their hard-earned qualifications cannot always shield them from prejudice and alienation may lead to a longing for home [62]. Wellbeing, thus, becomes an ideal which cannot be realized abroad without returning home physically or “socially.” In this regard, remittances and medical supplies sent home occasionally to assist family members or the community of origin should be read as social returns and symbolic efforts to allay the longing for home [63].

Although the findings highlighted in this paper shed some light into some of the dynamics of medical migration and some of the structural and psychosocial processes that can promote health personnel retention, this paper was neither intended to develop a full-fledged analysis of the Africa-to-USA physician brain drain, nor to draw policy implications from its findings. Doing so would have warranted one to address all potential questions the 36 main cells of the EPV matrix may raise. This represents a daunting task for a lone scholar (even one with broad-based knowledge) and is somewhat at odds with the interdisciplinary emphasis of the model. Fully interrogating all domains of capital of the EPV model at all levels of analysis and units of observation necessary entails a collaborative intellectual enterprise among scholars and practitioners from different disciplines but with shared interests in resolving outstanding problems. While resource-intensive and time-consuming, this inclusion of diverse perspectives and modes of inquiry in the analytic process ensures a critical dialogue about ideas and enhances the credibility and practicality of any policy informed by these findings [64].

Questions on the economic factors of health workforce migration and health personnel retention may be most appropriate for a research team led by economists with expertise in migration, development, and health financing. Political determinants of migration and personnel retention can be thoroughly scrutinized by political scientists, public administration scholars, and sociologists of migration. In-depth investigation of cultural determinants of health workforce migration would likely be most complete if led by anthropologists with subject matter expertise in migration, health, and development. Likewise, factors of migration and health personnel retention stemming from the physical and built environment, the purview of civil engineering, can best be addressed by scholars with expertise or working knowledge of civil engineering. Last, the analytical insight of psychologists could be critical for the exploration of personal level dynamics of migration.

As one of many frameworks available for interdisciplinary social research, the merit of the EPV model is its bringing to the fore the ubiquity of social power in human interactions. Whether conceived in terms of embodied capability, essence of the influencing process, or dynamic of social structures, power pervades human systems, and effects personal, organizational, and structural change [29, 65]. Power is wielded to oppress, and it is needed to resist, liberate, and empower, hence the CSDH’s recommendation for its equitable distribution among individuals and groups [55]. If knowledge and power are co-produced, physicians do have a good deal of both, at least in theory. The doctor-patient relationship is one of power asymmetry in favor of the doctor. Beyond the operating theater, physicians enjoy a space of privilege and symbolic capital in society very few professions enjoy [66]. Thus, while it may be reasonable to contend that many émigré physicians are mere victims of unfavorable socio-economic arrangements and political systems that compel them to leave their home countries, and that they would likely stay if the system was improved [67], it is unreasonable to discount physicians’ potentials as change agents of the systems change they wish to see.

Alternatively, there is an important distinction between health and healthcare, and doctors are mere “specialists in the narrow area of healthcare called medicine” [68]. Overstating their skills, talents, expertise, clout, capitals, professional obligations, social responsibility, and number as main remedies to failing health systems may prevent one from seeing the forest for the trees, and will take the focus away from important non-medical contextual factors that compound inequities in health and structure skilled migration as a whole. Werner, the author of the classic, *Where There is no Doctor*, states this fact eloquently: “No matter how many pediatricians are sent to Sierra Leone to tell a mother to feed her child, without any means, she will not follow their advice. Poverty is more powerful than knowledge” [68].

Poverty involves both material needs and capabilities deprivations, and while it is important to address both forms of deprivation, the latter is arguably more detrimental to health and human development than the former [69]. When the needs are many and the resources are limited, a multilevel framework such as the EPV model can help to identify the units of observation that require the most attention. The findings highlighted in this paper suggest that greater accountability in public administration and improvement of conditions of service in healthcare settings will be important remedies to the health personnel shortage in SSA. How such interventions may be achieved and sustained in the respective countries without involving some form of “violence” toward the beneficiaries of the current status quo is an open question. As Naim, reminds us, the most critical medicine in the therapy of a sick country is often the most elusive and random: the quality of its leadership [70].

## Conclusion

In a globalized, highly dynamic, and interconnected but greatly unequal world, tackling complex social and public health issues requires the use of comprehensive frameworks that capture the complexity of the interlocking environments that structure social problems. This paper sought to describe a transdisciplinary model grounded in ecological and psychopolitical thinking and potentially suitable for the comprehensive analysis of health personnel migration and other social determinants of health. Having been proposed for the first time in 2008 [29, 30], the EPV model is not new. However, it has barely been applied outside the interdisciplinary field of community psychology, and empirical validation of its potential for knowledge generation has yet to be fully investigated. This paper has suggested a way forward by highlighting how the EPV model can be applied to the collection and analysis of qualitative data in the social/behavioral sciences and in public health research.

## Footnotes

1 Atsu and Etse are aliases; these are colloquial names given to male twins in Ghana.
